# Supplementary material for: Learning non-stationary Langevin dynamics from stochastic observations of latent trajectories
Source: Nat Commun. 2021 Oct 13;12:5986. doi: 10.1038/s41467-021-26202-1 (PMC8514604; doi:10.1038/s41467-021-26202-1)
Supplement: Supplementary file 1 — Supplementary Information [file 41467_2021_26202_MOESM1_ESM.pdf]

Supplementary Information for: Learning non-stationary Langevin dynamics from stochastic observations of latent trajectories

Mikhail Genkin[1], Owen Hughes[2], and Tatiana A. Engel[1]
[1]Cold Spring Harbor Laboratory, Cold Spring Harbor, NY 11724
[2]University of Michigan, Ann Arbor, MI

Corresponding author e-mail: engel@cshl.edu

September 21, 2021

Contents

1 Supplementary Note 1: The likelihood calculation **2**

2 Supplementary Note 2: Variational derivatives of the likelihood **4**

3 Supplementary Note 3: Analytical derivation of the likelihood variational derivatives **5**

4 Supplementary Note 4: A relationship to the likelihood of an inhomogeneous Poisson process **9**

5 Supplementary Note 5: Derivation of the modified Fokker-Planck equation **10**

6 Supplementary Note 6: Feature complexity and model selection **12**
6.1 Feature complexity for non-stationary dynamics 12
6.2 Numerical calculation of feature complexity 14
6.3 Numerical calculation of the Jensen-Shannon divergence 15

7 Supplementary Table 1 . . . . **17**

8 Supplementary Figures **18**

9 Supplementary References **19**

1 Supplementary Note 1: The likelihood calculation

The model likelihood is given by Eq. (3), in which the transition probability densities $p(x_{t_i}|x_{t_{i-1}})$ are obtained from the solution of Eq. (6), and the probability densities of spike observations are $p(y_{t_i}|x_{t_i}) = f(x_{t_i})$. The absorption term $p(A|x_{t_E})$ only applies in the case of absorbing boundaries and is given by Eq. (9).

For an efficient numerical time-propagation of the latent probability density, we transform Eq. (6) to the Hermitian form with the operator $\boldsymbol{\mathcal{H}} = \exp(\Phi(x)/2)\hat{\boldsymbol{\mathcal{H}}}\exp(-\Phi(x)/2)$, where $\hat{\boldsymbol{\mathcal{H}}}$ is defined in Eq. (6) [1]. The Hermitian operator $\boldsymbol{\mathcal{H}}$ evolves the scaled probability density $\rho(x,t) = p(x,t)\exp(\Phi(x)/2)$ according to:

$$\frac{\partial\rho(x,t)}{\partial t} = -\boldsymbol{\mathcal{H}}\rho(x,t). \tag{1}$$

This operator consists of two parts $\boldsymbol{\mathcal{H}} = \boldsymbol{\mathcal{H}}_0 + \boldsymbol{\mathcal{H}}_I$, where $\boldsymbol{\mathcal{H}}_0$ accounts for the drift and diffusion in the latent space, and $\boldsymbol{\mathcal{H}}_I$ accounts for the decay of probability density due to spike emissions:

$$\begin{aligned}\boldsymbol{\mathcal{H}}_0 &= -e^{\Phi(x)/2}\frac{\partial}{\partial x}De^{-\Phi(x)}\frac{\partial}{\partial x}e^{\Phi(x)/2},\\ \boldsymbol{\mathcal{H}}_I &= f(x).\end{aligned} \tag{2}$$

We solve the eigenvector-eigenvalue problem for the Hermitian operator $\boldsymbol{\mathcal{H}}_0$:

$$\boldsymbol{\mathcal{H}}_0\Psi_0(x) = \lambda\Psi_0(x). \tag{3}$$

Using Supplementary Eq. (2), we rewrite Supplementary Eq. (3) as:

$$-\frac{\partial}{\partial x}De^{-\Phi(x)}\frac{\partial}{\partial x}\phi_0(x) = \lambda_0 e^{-\Phi(x)}\phi_0(x). \tag{4}$$

Here $\phi_0(x) = \exp(\Phi(x)/2)\Psi_0(x)$ are the scaled eigenfunctions corresponding to the scaled probability density $\rho(x,t)\exp(\Phi(x)/2) = p(x,t)\exp(\Phi(x))$. The eigenfunctions of the operator $\boldsymbol{\mathcal{H}}_0$ are obtained as $\Psi_0(x) = \phi_0(x)\exp(-\Phi(x)/2)$.

For the case of absorbing boundaries, $p(x,t)$ satisfies the forward Fokker-Planck equation with absorbing boundary conditions

$$[p(x,t)]_{x=\pm1} = 0, \tag{5}$$

and the scaled probability density $p(x,t)\exp(\Phi(x))$ satisfies the backward Fokker-Planck equation also with absorbing boundary conditions [1]. Thus the eigenfunctions $\phi_0(x)$ are obtained by solving Supplementary Eq. (4) with the boundary conditions $\phi_0(x)|_{x=\pm1} = 0$.

For the case of reflecting boundaries, $p(x,t)$ satisfies the Fokker-Planck equation with reflecting (zero flux) boundary conditions

$$\left[-\frac{d\Phi(x)}{dx}D(x)p(x,t) - D\frac{\partial p(x,t)}{\partial x}\right]_{x=\pm1} = 0, \tag{6}$$

and the scaled probability density satisfies the backward Fokker-Planck equation with zero derivative (Neumann) boundary conditions [1]. Thus the eigenfunctions $\phi_0(x)$ are obtained by solving Supplementary Eq. (4) with the boundary conditions $\partial\phi_0/\partial x|_{x=\pm1} = 0$.

To find the eigenfunctions of the operator $\boldsymbol{\mathcal{H}}$, we solve another eigenvalue-eigenvector problem $(\boldsymbol{\mathcal{H}}_0 + \boldsymbol{\mathcal{H}}_I)\Psi(x) = \lambda\Psi(x)$ in the basis of the operator $\boldsymbol{\mathcal{H}}_0$. As a result, we obtain an eigenbasis $\{\Psi_j(x)\}$ and eigenvalues $\{\lambda_j\}$ of the operator $\boldsymbol{\mathcal{H}}$, where $j = 1, 2, ...N_v$ and the eigenbasis is truncated to the N_v eigenvectors with the smallest eigenvalues. We refer to the operator $\boldsymbol{\mathcal{H}}$ as $\boldsymbol{\mathcal{H}}_{\text{abs}}$ or $\boldsymbol{\mathcal{H}}_{\text{ref}}$ for absorbing and reflecting boundary conditions, respectively.

We solve the eigenvector-eigenvalue problem Supplementary Eq. (4) using Spectral Elements Method (SEM) [2, 3, 4]. We use the Gauss-Legendre-Lobatto (GLL) grid with Lagrange interpolation polynomials as the basis functions (SEM basis). In the SEM basis, each function is represented by a vector that consists of the function values evaluated at the SEM grid points $\{x_i\}$, where $i = 1, 2, ..., N$. For example, the firing rate function $f(x)$ is represented by a vector $\boldsymbol{f}$ with components $f_i = f(x_i)$. In the SEM basis, the eigenbasis $\{\Psi_j(x)\}$ is a matrix $\boldsymbol{Q}$ of size $N \times N_v$ defined as $Q_{ij} = \Psi_j(x_i)$. The transformation between the SEM basis and the eigenbasis of $\boldsymbol{\mathcal{H}}$ is performed by generalized rules of basis transformations via $\tilde{\boldsymbol{f}} = \boldsymbol{Q^T W f}$ and $\boldsymbol{f} = \boldsymbol{Q}\tilde{\boldsymbol{f}}$, where $\tilde{\boldsymbol{f}}$ is the representation of $\boldsymbol{f}$ in the eigenbasis of $\boldsymbol{\mathcal{H}}$. Here $\boldsymbol{W} = \text{diag}(\boldsymbol{w})$ is the SEM weight matrix that normalizes the matrix of eigenvectors: $\boldsymbol{Q^T W Q} = \boldsymbol{I}$. Integration of functions in the SEM basis is performed with Gaussian quadrature by taking an inner product with the weight vector $\boldsymbol{w}$: $\int f(x)dx \approx \boldsymbol{f}^T\boldsymbol{w}$, where the weights for the GLL grid are calculated analytically [4].

We compute the likelihood in the eigenbasis of $\boldsymbol{\mathcal{H}}$. The formal solution of Supplementary Eq. (1) is given by

$$\rho(x_{t_k}|x_{t_{k-1}}) = e^{-\boldsymbol{\mathcal{H}}(t_k - t_{k-1})} \equiv e^{-\boldsymbol{\mathcal{H}}\Delta t_k}. \tag{7}$$

In the eigenbasis of $\boldsymbol{\mathcal{H}}$, the corresponding matrix is diagonal:

$$T_{k,ij} = \int_x \Psi_i(x) e^{-\boldsymbol{\mathcal{H}\Delta t_k}} \Psi_j(x) dx = \delta_{ij} e^{-\lambda_i \Delta t_k}. \tag{8}$$

In the $\boldsymbol{\mathcal{H}}$-basis, the spike emission operator matrix takes the form $\boldsymbol{E} = \boldsymbol{Q^T W f Q}$. The absorption operator takes the form $\boldsymbol{A} = \boldsymbol{Q}_{0H}^T \text{diag}(\boldsymbol{\lambda}_0)\boldsymbol{Q}_{0H}$, where λ_0 are the eigenvalues of $\boldsymbol{\mathcal{H}}_0$ obtained from Supplementary Eq. (3), and $\boldsymbol{Q}_{0H}$ is a transformation matrix from $\boldsymbol{\mathcal{H}}_0$-basis to $\boldsymbol{\mathcal{H}}$-basis. The integration over the terminal state x_{t_E} in Eq. (3) is realized by performing the inner product with a column vector $\boldsymbol{\beta}_{N+2} = \boldsymbol{Q}^T\boldsymbol{W}\boldsymbol{\rho}_{\mathbf{eq}}$, where $\boldsymbol{\rho}_{\mathbf{eq}}$ is a representation of the function $\exp(-\Phi(x)/2)$ in the $\boldsymbol{\mathcal{H}}$ basis. Since the operator $\boldsymbol{\mathcal{H}}$ propagates the scaled probability density $\rho(x,t)$, first scaling $\rho(x,t)$ with $\exp(-\Phi(x)/2)$ and then taking the inner product with the weight vector corresponds to the integration of the original probability density $p(x,t)$.

Thus, in the $\boldsymbol{\mathcal{H}}$ basis, the likelihood Eq. (3) is represented by a chain of matrix-vector multiplications:

$$\mathscr{L} = \boldsymbol{\rho}_0^T \boldsymbol{T}_1 \boldsymbol{E} \boldsymbol{T}_2 \boldsymbol{E} ... \boldsymbol{T}_N \boldsymbol{E} \boldsymbol{T}_{N+1} \boldsymbol{A} \boldsymbol{\beta}_{N+2}, \tag{9}$$

where the absorption operator $\boldsymbol{A}$ is applied only in the case of absorbing boundaries. Here $\boldsymbol{\rho}_0^T$ is a row vector that corresponds to the scaled initial probability density of latent states

$p_0(x)\exp(\Phi(x)/2)$ in the eigenbasis of the operator $\boldsymbol{\mathcal{H}}$. The chain of matrix-vector multiplications in Supplementary Eq. (9) realizes the integration over $x_{t_0}, x_{t_1}, ..., x_{t_N}$ in Eq. (3). The last inner product with the vector $\boldsymbol{\beta}_{N+2}$ realizes the integration over the final x_{t_E} in Eq. (3).

On each iteration of the gradient descent, we evaluate the likelihood numerically by calculating the eigenvalues and the eigenfunctions of the operator $\boldsymbol{\mathcal{H}}$, computing the matrices $\boldsymbol{T}_k$, $\boldsymbol{E}$, $\boldsymbol{A}$, and performing the chain of matrix-vector multiplications in Supplementary Eq. (9) from left to right (forward pass). To calculate the likelihood derivatives, we also perform a backward pass, where the chain in Supplementary Eq. (9) is calculated in the reverse order.

2 Supplementary Note 2: Variational derivatives of the likelihood

The variational derivatives of the model likelihood are obtained using calculus of variations as described previously [2]. The final analytical expressions read:

$$\begin{aligned}
\frac{\delta\mathscr{L}}{\delta F(x)} &= \sum_{ij} G_{ij}\frac{D}{2}e^{-\Phi(x)}\frac{d\left(\phi_i(x)\phi_j(x)\right)}{dx} + \frac{1}{2}\int_{-1}^{x}\left(\beta_0(s)p_0(s)e^{\Phi(s)/2} - \alpha_{N+2}(s)e^{-\Phi(s)/2}\right)ds,\\
\frac{\delta\mathscr{L}}{\delta F_0(x)} &= \int_{-1}^{x} p_0(s)\left(\mathscr{L} - e^{\Phi(s)/2}\beta_0(s)\right)ds, \qquad (10)\\
\frac{\partial\mathscr{L}}{\partial D} &= -\int_{-1}^{1} dx e^{-\Phi(x)}\sum_{ij} G_{ij}\frac{d\phi_i(x)}{dx}\frac{d\phi_j(x)}{dx}.
\end{aligned}$$

Here $F_0(x) = p_0'(x)/p(x)$ is an auxiliary function used to perform unconstrained optimization of $p_0(x)$ (Methods). $\phi_i(x) = \Psi_i(x)\exp(\Phi(x)/2)$ are the scaled eigenfunctions of $\boldsymbol{\mathcal{H}}$.

The functions $\alpha_i(x)$ and $\beta_i(x)$ are calculated with, respectively, the forward and backward passes through the chain Supplementary Eq. (9) in the eigenbasis of the operator $\boldsymbol{\mathcal{H}}$:

$$\begin{aligned}
&\boldsymbol{\alpha}_0 = \boldsymbol{\rho}_0^T,\\
&\boldsymbol{\alpha}_n = \boldsymbol{\alpha}_{n-1}\boldsymbol{T}_n\boldsymbol{E}, \qquad n = 1, 2\ldots N,\\
&\boldsymbol{\alpha}_{N+1} = \boldsymbol{\alpha}_N\boldsymbol{T}_{N+1}, \quad \boldsymbol{\alpha}_{N+2} = \boldsymbol{\alpha}_{N+1}\boldsymbol{A}. \qquad (11)
\end{aligned}$$

$$\begin{aligned}
&\boldsymbol{\beta}_{N+1} = \boldsymbol{A}\boldsymbol{\beta}_{N+2},\\
&\boldsymbol{\beta}_n = \boldsymbol{E}\boldsymbol{T}_{N+1}\boldsymbol{\beta}_{n+1}, \qquad n = 1, 2\ldots N,\\
&\boldsymbol{\beta}_0 = \boldsymbol{T}_1\boldsymbol{\beta}_1. \qquad (12)
\end{aligned}$$

The absorption matrix $\boldsymbol{A}$ in Supplementary Eqs. (11),(12) is applied only in the case of absorbing boundary conditions.

The matrix $\boldsymbol{G}$ is defined as:

$$G_{ij} = \sum_{\tau=0}^{N+1}\Gamma_{ij}^{\tau+1}\alpha_{\tau,i}\beta_{\tau+1,j}, \qquad (13)$$

where i, j index the components of the vectors $\boldsymbol{\alpha}_\tau$ and $\boldsymbol{\beta}_{\tau+1}$. The matrix $\boldsymbol{\Gamma}^\tau$ is defined for $\tau = N+2$ as $\boldsymbol{\Gamma}^\tau = -\boldsymbol{I}$, and for $\tau < N+2$ it is defined as:

$$\Gamma_{ij}^\tau = \int_0^{\Delta t_\tau} e^{-(\Delta t_\tau - u)\lambda_i} e^{-u\lambda_j} du = \begin{cases} \Delta t_\tau e^{-\lambda_i \Delta t_\tau}, & i = j, \\ \frac{e^{-\lambda_i \Delta t_\tau} - e^{-\lambda_j \Delta t_\tau}}{\lambda_j - \lambda_i}, & i \neq j, \end{cases} \tag{14}$$

where $\Delta t_\tau = t_\tau - t_{\tau-1}$ are interspike intervals, and λ_i are the eigenvalues of $\boldsymbol{\mathcal{H}}$. The vectors α_i are calculated with a forward pass Supplementary Eq. (11), and the vectors β_i and $\boldsymbol{G}$ are subsequently calculated with a backward pass Supplementary Eqs. (12)-(14). The implementation of the forward and backward passes is based on the sum-product and scaling algorithms, similar to the Hidden Markov Models [5]. The algorithm scales all $\boldsymbol{\alpha}_i$ and $\boldsymbol{\beta}_j$ so that $\alpha_i^T \beta_i = 1$ for all i, which prevents numerical underflow. The antiderivatives (indefinite integrals) in Supplementary Eq. (10) are evaluated by multiplication with the integration matrix that is constructed by analytical integration of the Lagrange interpolation polynomials [2].

While we perform computations in the SEM basis, the likelihood derivatives Supplementary Eq. (10) are expressed in terms of continuous functions, which can be discretized in any appropriate basis with arbitrary precision specified by the basis dimensionality. Detailed derivation of Supplementary Eq. (10) is provided in Supplementary Information 3.

3 Supplementary Note 3: Analytical derivation of the likelihood variational derivatives

Here we derive the analytical expressions Supplementary Eq. (10). For convenience, we represent the likelihood using the Dirac bra-ket notation [2, 3]:

$$\mathscr{L}\left[Y(t)|\theta\right] = \left\langle \rho_0 \middle| e^{-\boldsymbol{\mathcal{H}}(t_1 - t_0)} \boldsymbol{y}_{t_1} e^{-\boldsymbol{\mathcal{H}}(t_2 - t_1)} \boldsymbol{y}_{t_2} ... e^{-\boldsymbol{\mathcal{H}}(t_N - t_{N-1})} \boldsymbol{y}_{t_N} e^{-\hat{\boldsymbol{\mathcal{H}}}(t_E - t_N)} \boldsymbol{A} \middle| \beta_{N+2} \right\rangle. \tag{15}$$

In this notation, the scaled latent probability density $\rho(x,t) = p(x,t)\exp(\Phi(x)/2)$ is represented by the bra vectors, with the initial state $\langle \rho_0 |$. The time-propagation of the latent probability density is carried out by the operator $\exp(-\boldsymbol{\mathcal{H}}\Delta t_\tau)$, where $\Delta t_\tau = t_\tau - t_{\tau-1}$. The operators $\boldsymbol{y}_{t_\tau}$ account for the observed spikes. The ket $|\beta_{N+2}\rangle$ accounts for the marginalization over the terminal latent state x_{t_E}.

To find variational derivative of the likelihood with respect to the force $F(x)$, we differentiate Supplementary Eq. (15) applying the product rule of derivatives. The likelihood dependence on $F(x)$ is hidden inside the terms ρ_0 and β_{N+2}, and in each of the operators $\exp(-\boldsymbol{\mathcal{H}}\Delta t_\tau)$, and $\boldsymbol{A}$. Thus, the derivative is given by the sum:

$$\begin{aligned} \frac{\delta \mathscr{L}}{\delta F(x)} = &\sum_{i,j} \left[\sum_{\tau=1}^{N+1} a_i(\tau-1) b_j(\tau) \frac{\delta \langle \Psi_i | e^{-\boldsymbol{\mathcal{H}}\Delta t_\tau} | \Psi_j \rangle}{\delta F} + a_i(N+1) b_j(N+2) \frac{\delta \langle \Psi_i | \boldsymbol{A} | \Psi_j \rangle}{\delta F} \right] + \\ &+ \sum_i \left[\frac{\delta a_i(0)}{\delta F} b_i(0) + a_i(N+2) \frac{\delta b_i(N+2)}{\delta F} \right] \equiv L_1 + L_2, \end{aligned} \tag{16}$$

where i and j index the eigenfunctions of the operator $\boldsymbol{\mathcal{H}}$. In Supplementary Eq. (16), we introduced the quantities:

$$\begin{aligned}&\langle\alpha_0| = \langle\rho_0|, \qquad \langle\alpha_n| = \langle\alpha_{n-1}|e^{-\boldsymbol{\mathcal{H}}\Delta t_n}\boldsymbol{y}_{t_n},\ n = 1, 2\dots N,\\&\langle\alpha_{N+1}| = \langle\alpha_N|e^{-\boldsymbol{\mathcal{H}}\Delta t_{N+1}}, \qquad \langle\alpha_{N+2}| = \langle\alpha_{N+1}|\boldsymbol{A},\\&|\beta_{N+1}\rangle = \boldsymbol{A}|\beta_{N+2}\rangle,\\&|\beta_n\rangle = \boldsymbol{y}_{t_n}e^{-\boldsymbol{\mathcal{H}}\Delta t_{n+1}}|\beta_{n+1}\rangle,\ n = 1, 2, \dots N, \qquad |\beta_0\rangle = e^{-\boldsymbol{\mathcal{H}}\Delta t_1}|\beta_1\rangle,\\&a_i(\tau) = \langle\alpha_\tau|\Psi_i\rangle, \qquad b_j(\tau) = \langle\Psi_j|\beta_\tau\rangle.\end{aligned} \tag{17}$$

In a finite basis, such as the truncated eigenbasis of the operator $\boldsymbol{\mathcal{H}}$, each ket $\langle\alpha_\tau|$ corresponds to a vector $\boldsymbol{\alpha}_\tau$, and each $a_i(\tau)$ is the i-th entry of this vector, compare Supplementary Eq. (17) with Supplementary Eqs. (11),(12). Thus, $a_i(\tau)$ and $b_i(\tau)$ are calculated with the forward-backward pass.

We first derive the expression for the first term in Supplementary Eq. (16) denoted as L_1. Using the formula for the derivative of an exponential operator [6], we obtain [2, 3]:

$$\frac{\delta\langle\Psi_i|e^{-\boldsymbol{\mathcal{H}}\Delta t_\tau}|\Psi_j\rangle}{\delta F} = -\left\langle\Psi_i\middle|\frac{\partial\boldsymbol{\mathcal{H}}}{\partial F}\middle|\Psi_j\right\rangle\Gamma^\tau_{i,j}, \tag{18}$$

where the matrix $\boldsymbol{\Gamma}^\tau$ is defined in Supplementary Eq. (14), and here $\tau = 1, 2, \dots N+1$. The operator $\boldsymbol{\mathcal{H}}$ can be written as [3]:

$$\boldsymbol{\mathcal{H}} = -D\nabla^2 + D\frac{F'(x)}{2} + D\frac{F^2(x)}{4} + f(x). \tag{19}$$

Using the Euler-Lagrange equation for a variational derivative, we obtain:

$$\begin{aligned}\left\langle\Psi_i\middle|\frac{\delta\boldsymbol{\mathcal{H}}}{\delta F}\middle|\Psi_j\right\rangle &= \frac{\delta}{\delta F}D\int\Psi_i(x)\left(\frac{F'(x)}{2} + \frac{F(x)^2}{4}\right)\Psi_j(x)dx =\\ \frac{D}{2}\left(F(x)\Psi_i(x)\Psi_j(x) - \frac{d\left(\Psi_i(x)\Psi_j(x)\right)}{dx}\right) &= -\frac{D}{2}\exp(-\Phi(x))\frac{d(\phi_i(x)\phi_j(x))}{dx}.\end{aligned} \tag{20}$$

Here we normalize $\Phi(x)$ such that $\int\exp(-\Phi(x))dx = 1$, and $\phi_i(x) = \Psi_i(x)\exp(\Phi(x)/2)$ are the scaled eigenfunctions.

The absorption operator is $\boldsymbol{A} = \boldsymbol{\mathcal{H}}_0$, where $\boldsymbol{\mathcal{H}}_0$ is the part of $\boldsymbol{\mathcal{H}}$ that accounts for the drift and diffusion in the latent space:

$$\boldsymbol{\mathcal{H}}_0 = -D\nabla^2 + D\frac{F'(x)}{2} + D\frac{F^2(x)}{4}. \tag{21}$$

Therefore, the derivative of the operator $\boldsymbol{A}$ with respect to $F(x)$ is the same as the derivative of the operator $\boldsymbol{\mathcal{H}}$ and is given by Supplementary Eq. (20).

Combining Supplementary Eqs. (18),(20), we obtain the expression for L_1 (c.f. with the first term in Supplementary Eq. (10)):

$$L_1 = \sum_{ij}G_{ij}\frac{D}{2}e^{-\Phi(x)}\frac{d\left(\phi_i(x)\phi_j(x)\right)}{dx}. \tag{22}$$

Here the matrix $\boldsymbol{G}$, defined in Supplementary Eq. (13), gathers the contributions from all terms $\exp(-\boldsymbol{\mathcal{H}}\Delta t_i)$ and $\boldsymbol{A}$ (where for $\tau = N+2$ we define $\boldsymbol{\Gamma}^\tau = -\boldsymbol{I}$, so that Supplementary Eq. (18) also holds for the operator $\boldsymbol{A}$).

Next, we compute the second term in Supplementary Eq. (16) denoted as L_2, which requires calculating the derivatives of $a_i(0)$ and $b_i(N+2)$ with respect to $F(x)$. For convenience, we write this term in the function representation:

$$\begin{aligned} L_2 = \sum_i \left[\frac{\delta a_i(0)}{\delta F} b_i(0) + a_i(N+2)\frac{\delta b_i(N+2)}{\delta F}\right] = \\ = \frac{\delta}{\delta F(x)}\left[\int_{-1}^{1}\alpha_0(x)\beta_0(x)dx + \int_{-1}^{1}\alpha_{N+2}(x)\beta_{N+2}(x)dx\right] \equiv \frac{\delta}{\delta F(x)}\left[l_1 + l_2\right]. \end{aligned} \tag{23}$$

Here the functions $\alpha_i(x)$ and $\beta_i(x)$ correspond to $\langle\alpha_i|$ and $|\beta_i\rangle$ in Supplementary Eq. (17), respectively. We have $\alpha_0(x) = p_0(x)\exp(\Phi(x)/2)$, $\beta_{N+2}(x) = \exp(-\Phi(x)/2)$ (Methods), and the derivative does not apply to the functions $\beta_0(x)$ and $\alpha_{N+2}(x)$, since their dependence on $F(x)$ is already accounted for in the term L_1 in Supplementary Eq. (16). The derivatives are evaluated using the chain rule for functional derivatives:

$$\frac{\delta l_1}{\delta F(x)} = \int_{-1}^{1} ds \frac{\delta l_1}{\delta \Phi(s)}\frac{\delta \Phi(s)}{\delta F(x)}, \tag{24}$$

where $l_1 = \int_{-1}^{1} p_0(x)\exp(\Phi(x)/2)\beta_0(x)dx$. Using the Euler-Lagrange equation, we obtain:

$$\begin{aligned} \frac{\delta l_1}{\delta \Phi(s)} &= \frac{1}{2}p_0(s)\exp(\Phi(s)/2)\beta_0(s), \\ \frac{\delta \Phi(s)}{\delta F(x)} &= -\frac{\delta}{\delta F(x)}\int_{-1}^{s} F(s')ds' = -\frac{\delta}{\delta F(x)}\int_{-1}^{1} F(s')H(s-s')ds' = \\ &= -H(s-x) = -1 + H(x-s), \end{aligned} \tag{25}$$

where H is the Heaviside step function. Thus, we obtain:

$$\begin{aligned} \frac{\delta l_1}{\delta F(x)} &= \int_{-1}^{1} ds \frac{1}{2}p_0(s)\exp(\Phi(s)/2)\beta_0(s)(-1 + H(x-s)) = \\ &= -\frac{\mathscr{L}}{2} + \frac{1}{2}\int_{-1}^{x} ds p_0(s)\exp(\Phi(s)/2)\beta_0(s). \end{aligned} \tag{26}$$

The variational derivative of the second term l_2 in Supplementary Eq. (23) is calculated similarly. As a result, we obtain the expression for L_2 (c.f. with the second term in Supplementary Eq. (10)):

$$L_2 = \frac{1}{2}\int_{-1}^{x} ds \left(p_0(s)\exp(\Phi(s)/2)\beta_0(s) - a_{N+2}(s)\exp(-\Phi(s)/2)\right). \tag{27}$$

To derive the expression for $\delta\mathscr{L}/\delta F_0(x)$, we write the likelihood in the function representation:

$$\mathscr{L} = \int_{-1}^{1} p_0(x)\exp(\Phi(x)/2)\beta_0(x)dx. \tag{28}$$

The likelihood depends on $F_0(x)$ only through the term $p_0(x)$:

$$\frac{\delta \mathscr{L}}{\delta F_0(x)} = \int_{-1}^{1} ds \frac{\delta \mathscr{L}}{\delta p_0(s)} \frac{\delta p_0(s)}{\delta F_0(x)}. \tag{29}$$

We compute the derivative $\delta \mathscr{L}/\delta p_0[s]$ using the Euler-Lagrange equation:

$$\frac{\delta \mathscr{L}}{\delta p_0(s)} = \exp(\Phi(s)/2)\beta_0(s). \tag{30}$$

To compute the derivative $\delta p_0[s]/\delta F_0(x)$, we express $p_0(x)$ through $F_0(x)$ (Methods):

$$p_0(x) = \frac{\exp(\int_{-1}^{x} F_0(x')dx')}{\int_{-1}^{1} \exp(\int_{-1}^{x} F_0(x')dx')dx} \equiv \frac{e^{G(x)}}{\int_{-1}^{1} e^{G(x')}dx'} = \frac{\int_{-1}^{1} e^{G(x')}\delta(x-x')dx'}{\int_{-1}^{1} e^{G(x')}dx'}. \tag{31}$$

Here $G(x) = \int_{-1}^{1} F_0(x')H(x-x')dx'$, and H is the Heaviside step function. Using the chain rule, we obtain:

$$\frac{\delta p_0(s)}{\delta F_0(x)} = \int_{-1}^{1} ds' \frac{\delta p_0(s)}{\delta G(s')} \frac{\delta G(s')}{\delta F_0(x)}. \tag{32}$$

Using the Euler-Lagrange equation, we get:

$$\begin{aligned} \frac{\delta p_0(s)}{\delta G(s')} &= \frac{e^{G(s')}\delta(s-s') \int_{-1}^{1} e^{G(x')}dx' - e^{G(s')}e^{G(s)}}{\left(\int_{-1}^{1} e^{G(x')}dx'\right)^2}, \\ \frac{\delta G(s')}{\delta F(x)} &= H(s'-x). \end{aligned} \tag{33}$$

As a result,

$$\begin{aligned} \frac{\delta p_0(s)}{\delta F_0(x)} &= \int_{-1}^{1} ds' \left[\frac{e^{G(s')}\delta(s-s')}{\int_{-1}^{1} e^{G(x')}dx'} - \frac{e^{G(s')}e^{G(s)}}{\left(\int_{-1}^{1} e^{G(x')}dx'\right)^2} \right] H(s'-x) = \\ &= \frac{e^{G(s)}}{\int_{-1}^{1} e^{G(x')}dx'} \left[H(s-x) - \frac{\int_{-1}^{1} e^{G(s')}H(s'-x)ds'}{\int_{-1}^{1} e^{G(x')}dx'} \right] = \\ &= p_0(s) \left[H(s-x) - \frac{\int_{x}^{1} e^{G(s')}ds'}{\int_{-1}^{1} e^{G(x')}dx'} \right] = \\ &= p_0(s) \left[H(s-x) - \int_{x}^{1} p_0(s')ds' \right] = p_0(s) \left[-H(x-s) + \int_{-1}^{x} p_0(s')ds' \right]. \end{aligned} \tag{34}$$

By substituting Supplementary Eqs. (34),(30) into Supplementary Eq. (29) we obtain:

$$\begin{aligned} \frac{\delta \mathscr{L}}{\delta F_0(x)} &= \int_{-1}^{1} ds \exp(\Phi(s)/2)\beta_0(s)p_0(s) \left[-H(x-s) + \int_{-1}^{x} p_0(s')ds' \right] = \\ &= -\int_{-1}^{x} ds \exp(\Phi(s)/2)\beta_0(s)p_0(s) + \mathscr{L} \int_{-1}^{x} p_0(s')ds' = \int_{-1}^{x} p_0(s)\left(\mathscr{L} - \exp(\Phi(s)/2)\beta_0(s)\right) ds. \end{aligned} \tag{35}$$

Finally, we derive the expression for the derivative $d\mathscr{L}/dD$. The likelihood Supplementary Eq. (15) depends on D through the operators $\exp(-\boldsymbol{\mathcal{H}}\Delta t_\tau)$ and $\boldsymbol{A}$. Applying the product rule for derivatives, we obtain:

$$\frac{\partial \mathscr{L}}{\partial D} = \sum_{i,j} \left[\sum_{\tau=1}^{N+1} a_i(\tau-1) b_j(\tau) \frac{\partial \langle \Psi_i | e^{-\boldsymbol{\mathcal{H}}\Delta t_\tau} | \Psi_j \rangle}{\partial D} + a_i(N+1) b_j(N+2) \frac{\partial \langle \Psi_i | \boldsymbol{A} | \Psi_j \rangle}{\partial D} \right], \tag{36}$$

where $a_i(\tau)$, $b_j(\tau)$ are defined in Supplementary Eq. (17). Similarly to Supplementary Eq. (18), we have

$$\frac{\partial \langle \Psi_i | e^{-\boldsymbol{\mathcal{H}}\Delta t_\tau} | \Psi_j \rangle}{\partial D} = -\left\langle \Psi_i \middle| \frac{\partial \boldsymbol{\mathcal{H}}}{\partial D} \middle| \Psi_j \right\rangle \Gamma^\tau_{i,j}, \tag{37}$$

where the matrix Γ^τ is defined in Supplementary Eq. (14). Using Supplementary Eq. (19) and the Euler-Lagrange equation, we obtain:

$$\begin{aligned} \left\langle \Psi_i \middle| \frac{d\boldsymbol{\mathcal{H}}}{dD} \middle| \Psi_j \right\rangle = \int_{-1}^{1} \left[\Psi_i(x) \left(\frac{F'(x)}{2} + \frac{F^2(x)}{4} \right) \Psi_j(x) - \Psi_i(x) \Psi_j''(x) \right] dx = \\ - \int_{-1}^{1} \left[\phi_i(x) \left(\exp(-\Phi(x)) \phi_j'(x) \right)' \right] dx = \int_{-1}^{1} \exp(-\Phi(x)) \phi_i'(x) \phi_j'(x) dx, \end{aligned} \tag{38}$$

where we used integration by parts, and the boundary term vanishes for both absorbing and reflecting boundary conditions.

Combining Supplementary Eqs. (36),(37),(38), we obtain (c.f. Supplementary Eq. (16)):

$$\frac{\partial \mathscr{L}}{\partial D} = -\int_{-1}^{1} dx e^{-\Phi(x)} \sum_{ij} G_{ij} \phi_i'(x) \phi_j'(x), \tag{39}$$

where the matrix $\boldsymbol{G}$ is defined by Supplementary Eq. (13).

4 Supplementary Note 4: A relationship to the likelihood of an inhomogeneous Poisson process

In this section we derive the likelihood of a spike sequence $Y(t)$ from an inhomogeneous Poisson process when the latent trajectory $\mathcal{X}(t)$ is fixed and fully observed. The data $Y(t) = \{t_0, t_1, ..., t_N, t_E\}$ consist of spike times $t_1, t_2, \dots t_N$, and t_0 and t_E are the trial start and end times, respectively. Since the trajectory is fully observed, the time-dependent firing rate of the Poisson process $\lambda(t)$ is known: $\lambda(t) = f(\mathcal{X}(t))$, where $f(x)$ is the firing-rate function. By definition of the Poisson firing rate, the probability density $p(y_{t_i})$ of a spike occurring at time t_i equals $\lambda(t_i)$, and the probability of not observing a spike in a time interval $[t_{i-1}; t_i]$ is given by [7]:

$$\bar{p}(t_i, t_{i-1}) = P(\text{no spikes in } [t_{i-1}; t_i]) = \exp\left(-\int_{t_{i-1}}^{t_i} \lambda(t) dt \right). \tag{40}$$

Since Poisson process has no memory, the probability density of observing the entire spike sequence $Y(t)$ is a product of the probability densities of all observed spikes and probabilities of not observing a spike during all interspike intervals (cf. Eq. (4)):

$$p(Y(t)|\lambda(t)) = \left[\prod_{i=1}^{N} p(y_{t_i})\bar{p}(t_i, t_{i-1})\right] \bar{p}(t_E, t_N). \tag{41}$$

Substituting Supplementary Eq. (40) and $p(y_{t_i}) = \lambda(t_i)$ into Supplementary Eq. (41), we obtain:

$$p\left(Y(t)|\lambda(t)\right) = \left[\prod_{i=1}^{N} \lambda(t_i) \exp\left(-\int_{t_{i-1}}^{t_i} \lambda(t)dt\right)\right] \exp\left(-\int_{t_N}^{t_E} \lambda(t)dt\right). \tag{42}$$

Rearranging the terms in Supplementary Eq. (42) to combine all exponents into a single exponent, we obtain a familiar equation for the likelihood of a spike train from an inhomogeneous Poisson process with a known time-dependent firing rate $\lambda(t)$ [7]:

$$p\left(Y(t)|\lambda(t)\right) = \exp\left(-\int_{t_0}^{t_E} \lambda(t)dt\right) \prod_{i=1}^{N} \lambda(t_i). \tag{43}$$

Thus, for a single fully observed trajectory $\mathcal{X}(t)$, the likelihood of spike data $Y(t)$ is simply given by the likelihood of an inhomogeneous Poisson process. Supplementary Eq. (43) is widely used for maximum likelihood estimation of parameters in neural encoding models, in which the relationship between a stimulus and firing rate is implemented by a known parametric function [8].

5 Supplementary Note 5: Derivation of the modified Fokker-Planck equation

Our goal is to infer latent Langevin dynamics from spike data. In this case, the one specific trajectory that produced the observed spike sequence is unknown and therefore we cannot directly use the likelihood of an inhomogeneous Poisson process Supplementary Eq. (43). Instead, we have to consider all possible latent trajectories that may have produced the data $Y(t)$ and weight each trajectory not only according to how consistent it is with the data (Supplementary Eq. (43)) but also how likely it can arise from the Langevin dynamics.

To perform this computation, we again use the fact that Poisson process has no memory and factorize the probability density of the entire spike sequence into probability densities of observing each spike and probabilities of not observing a spike during interspike intervals, as in Supplementary Eq. (41). We denote the latent state at each of the observation times $\{t_0, t_1, \dots, t_N, t_E\}$ by $X(t) = \{x_{t_0}, x_{t_1}, \dots, x_{t_N}, x_{t_E}\}$, which is a discrete set of points along a continuous path $\mathcal{X}(t)$. We fix the set of points $X(t)$ and later marginalize over it in Eq. (3).

Fixing the latent state x_{t_i} at the spike time t_i allows us to find the probability density of this spike occurring $p(y_{t_i}|x_{t_i}) = f(x(t_i))$, the same way as we did in Supplementary Eq. (42).

To compute the probability of no spikes occurring during the interspike interval $[t_{i-1}; t_i]$, we need to average the probability in Supplementary Eq. (40) over all possible latent paths that connect $x_{t_{i-1}}$ and x_{t_i}. This averaging corresponds to the expectation of the stochastic path integral [3]:

$$p(x_{t_i}|x_{t_{i-1}}) = \mathop{\mathbb{E}}_{\mathcal{X}(t)}\left[\exp\left(-\int_{t_{i-1}}^{t_i} f(\mathcal{X}(t))dt\right)\delta\left(\mathcal{X}(t_i) - x_{t_i}\right)\middle|\mathcal{X}(t_{i-1}) = x_{t_{i-1}}\right]. \tag{44}$$

Here a stochastic trajectory $\mathcal{X}(t)$ follows the Langevin dynamics Eq. (1), with the fixed start and end points: the state x_{t_i} at time t_i and the state $x_{t_{i-1}}$ at time t_{i-1}. Using the Feynman-Kac formula, the path integral Supplementary Eq. (44) can be transformed into the backward Kolmogorov equation [9]:

$$\begin{aligned}\frac{\partial p(x,t|x',t')}{\partial t'} &= \left(-DF(x')\frac{\partial}{\partial x'} - D\frac{\partial^2}{\partial x'^2} + f(x')\right)p(x,t|x',t') \equiv \hat{\boldsymbol{\mathcal{H}}}^{\dagger} p(x,t|x',t'),\\ p(x,t|x',t) &= \delta(x-x')\end{aligned} \tag{45}$$

To find the corresponding forward equation, we derive the adjoint operator:

$$\begin{aligned}\langle h(x), \hat{\boldsymbol{\mathcal{H}}}^{\dagger} q(x)\rangle = \int h(x)\left(-DF(x)\frac{\partial}{\partial x} - D\frac{\partial^2}{\partial x^2} + f(x)\right)q(x)dx = \\ \int\left[\left(\frac{\partial}{\partial x}DF(x) - \frac{\partial^2}{\partial x^2}D + f(x)\right)h(x)\right]q(x)dx + Q(h,q) = \langle\hat{\boldsymbol{\mathcal{H}}}h(x), q(x)\rangle + Q(h,q).\end{aligned} \tag{46}$$

Here $Q(h, q)$ is a boundary term that arises from integration by parts. For both the reflecting and absorbing boundary conditions for the function $h(x)$ (in the forward equation), the boundary conditions for the function $q(x)$ (in the backward equation) are chosen so that the boundary term is zero [1]. The operator $\hat{\boldsymbol{\mathcal{H}}}$ in Supplementary Eq. (46) contains the usual drift and diffusion terms that are the same as in the Fokker-Planck operator for the Langevin dynamics Eq. (1). In addition, it contains the term $f(x)$ that accounts for the decay of the probability density due to spike emissions through the inhomogeneous Poisson process. We refer to the forward equation with the operator $-\hat{\boldsymbol{\mathcal{H}}}$ as a modified Fokker-Planck equation:

$$\frac{\partial p(x,t|x',t')}{\partial t} = -\hat{\boldsymbol{\mathcal{H}}}p(x,t|x',t') = \left(-D\frac{\partial}{\partial x}F(x) + D\frac{\partial^2}{\partial x^2} - f(x)\right)p(x,t|x',t'). \tag{47}$$

We use this equation to propagate the latent probability density between the adjacent spikes, which is the same as computing the probability of not observing spikes during interspike intervals averaged over all possible latent paths connecting the states $x_{t_{i-1}}$ and x_{t_i}. In the main text Eq. (6), we use $p(x, t)$ instead of $p(x, t|x', t')$ to keep the notation simple.

6 Supplementary Note 6: Feature complexity and model selection

6.1 Feature complexity for non-stationary dynamics

Feature complexity is the negative entropy of latent trajectories. We derive an analytical expression Eq. (14) for the trajectory entropy Eq. (13) for non-equilibrium dynamics following the steps similar to the derivation of the equilibrium trajectory entropy [10]. First, we discretize time with a uniform step Δt and represent a continuous latent trajectory as a sequence of states $X = [x_0, x_1, x_2, ...x_N]$, so that Eq. (13) turns into:

$$S = -\lim_{\Delta t \to 0} \int dx_0 dx_1 \dots dx_N P(X) \ln \frac{P(X)}{Q(X)} \tag{48}$$

Using the Markov property of Langevin dynamics, we substitute the factorizations $P(X) = p(x_0) \prod_\tau p(x_{\tau+1}|x_\tau)$ and $Q(X) = q(x_0) \prod_\tau q(x_{\tau+1}|x_\tau)$ into Supplementary Eq. (48):

$$S = -\lim_{\Delta t \to 0} \int \left(\prod_{\tau'=0}^{N-1} dx_{\tau'} dx_N p(x_0) p(x_{\tau'+1}|x_{\tau'}) \right) \left(\ln \frac{p(x_0)}{q(x_0)} + \sum_{\tau=0}^{N-1} \ln \frac{p(x_{\tau+1}|x_\tau)}{q(x_{\tau+1}|x_\tau)} \right). \tag{49}$$

This expression can be reorganized by interchanging the integration order:

$$\begin{aligned} S = -\lim_{\Delta t \to 0} \sum_{\tau=0}^{N-1} \int dx_{\tau+1} dx_\tau p(x_{\tau+1}|x_\tau) \ln \frac{p(x_{\tau+1}|x_\tau)}{q(x_{\tau+1}|x_\tau)} \times \\ \left[\int \prod_{\substack{\tau' \neq \tau \\ \tau' \neq \tau+1}}^{N-1} dx_{\tau'} dx_N p(x_0) p(x_{\tau'+1}|x_{\tau'}) p(x_{\tau+2}|x_{\tau+1}) \right] - \\ \int dx_0 \left[\int \prod_{\tau'=0}^{N-1} dx_{\tau'+1} p(x_0) p(x_{\tau'+1}|x_{\tau'}) \right] \ln \frac{p(x_0)}{q(x_0)}. \end{aligned} \tag{50}$$

The terms in square brackets can be simplified using the normalization property of probability densities:

$$\begin{aligned} &\int \prod_{\substack{\tau' \neq \tau \\ \tau' \neq \tau+1}}^{N-1} dx_{\tau'} dx_N p(x_0) p(x_{\tau'+1}|x_{\tau'}) p(x_{\tau+2}|x_{\tau+1}) = \\ &= \left(\int dx_{\tau+2} p(x_{\tau+2}|x_{\tau+1}) \cdots \left(\int dx_{N-1} p(x_{N-1}|x_{N-2}) \left(\int dx_N p(x_N|x_{N-1}) \right) \right) \cdots \right) \times \\ &\times \left(\int dx_{\tau-1} p(x_\tau|x_{\tau-1}) \cdots \left(\int dx_1 p(x_2|x_1) \left(\int dx_0 p(x_1|x_0) p(x_0) \right) \right) \cdots \right) = p(x_\tau). \end{aligned} \tag{51}$$

$$
\int \prod_{\tau'=0}^{N-1} dx_{\tau'+1} p(x_0) p(x_{\tau'+1}|x_{\tau'}) = p(x_0)\times
\left(\int dx_1 p(x_1|x_0) \cdots \left(\int dx_{N-1} p(x_{N-1}|x_{N-2}) \left(\int dx_N p(x_N|x_{N-1})\right)\right) \cdots \right) = p(x_0). \tag{52}
$$

Substituting Supplementary Eqs. (51), (52) into Supplementary Eq. (50), we obtain:

$$
S = -\lim_{\Delta t\to 0} \sum_{\tau=0}^{N-1} \int dx_\tau dx_{\tau+1} p(x_{\tau+1}, x_\tau) \ln \frac{p(x_{\tau+1}|x_\tau)}{q(x_{\tau+1}|x_\tau)} - \int dx_0 p(x_0) \ln \frac{p(x_0)}{q(x_0)}. \tag{53}
$$

We introduce the following notation:

$$
S_{\text{eq}} = -\int dx_0 p(x_0) \ln \frac{p(x_0)}{q(x_0)}, \tag{54}
$$

$$
S_{\text{KL}}(\Delta t, \tau) = -\int dx_\tau dx_{\tau+1} p(x_{\tau+1}, x_\tau) \ln \frac{p(x_{\tau+1}|x_\tau)}{q(x_{\tau+1}|x_\tau)}. \tag{55}
$$

Now we can rewrite Supplementary Eq. (53) as:

$$
S = S_{\text{eq}} + \lim_{\Delta t\to 0} \sum_\tau S_{\text{KL}}(\Delta t, \tau) = S_{\text{eq}} + \int_0^\infty d\tau \lim_{\Delta t\to 0} \frac{S_{\text{KL}}(\Delta t, \tau)}{\Delta t}. \tag{56}
$$

Following Ref. [10], we evaluate the limit in the second term with a L'Hopitals rule (for notation convenience, we now use the subscript in x_τ to represent the continuous time variable):

$$
\lim_{\Delta t\to 0} \frac{S_{\text{KL}}(\Delta t, \tau)}{\Delta t} = -\left.\frac{\partial \left(\int dx_\tau dx_{\tau+t} p(x_{\tau+t}, x_\tau) \ln \frac{p(x_{\tau+t}|x_\tau)}{q(x_{\tau+t}|x_\tau)}\right)}{\partial t}\right|_{t=0} =
= -\mathop{\mathbb{E}}_{p(x_\tau)} \left[\frac{\partial}{\partial t} \left.\left(\int dx_{t+\tau} p(x_{\tau+t}|x_\tau) \ln \frac{p(x_{\tau+t}|x_{\tau)}}{q(x_{\tau+t}|x_\tau)}\right)\right|_{t=0} \right]. \tag{57}
$$

Interchanging integration with the time derivative, we obtain:

$$
\lim_{\Delta t\to 0} \frac{S_{\text{KL}}(\Delta t, \tau)}{\Delta t} = -\int dx_\tau p(x_\tau) dx_{\tau+t}
\left.\left[\partial_t p(x_{\tau+t}|x_\tau) \ln \frac{p(x_{\tau+t}|x_\tau)}{q(x_{\tau+t}|x_\tau)} + \partial_t p(x_{\tau+t}|x_\tau) - \frac{p(x_{\tau+t}|x_\tau)}{q(x_{\tau+t}|x_\tau)} \partial_t q(x_{\tau+t}|x_\tau)\right]\right|_{t=0}. \tag{58}
$$

To evaluate the expression in square brackets, we use the asymptotic solution for $p(x_{\tau+t}|x_\tau)$ valid for small times t [11]:

$$
p(x_{\tau+t}|x_\tau) = \frac{1}{2\sqrt{\pi D t}} \exp\left[-\frac{(x_{\tau+t} - x_\tau - DF(x_\tau)t)^2}{4Dt}\right] + O(t^2), \tag{59}
$$

where $F(x)$ is the driving force in the Langevin equation. For the reference model (free diffusion with zero driving force), the exact analytical solution for $q(x_{\tau+t}|x_\tau)$ is:

$$q(x_{\tau+t}|x_\tau) = \frac{1}{2\sqrt{\pi D t}} \exp\left[-\frac{(x_{\tau+t} - x_\tau)^2}{4Dt}\right], \tag{60}$$

which is valid for all times. Here we also took into account that the reference model has the same diffusion coefficient D. Taking the time derivatives in Supplementary Eqs. (59), (60), we obtain:

$$\partial_t p(x_{\tau+t}|x_\tau) = \frac{1}{2\sqrt{\pi D t}} \exp\left[-\frac{(x_{\tau+t} - x_\tau - DF(x_\tau)t)^2}{4Dt}\right] \left(-\frac{1}{2t} - \frac{DF^2(x_\tau)}{4} + \frac{(x_{\tau+t} - x_\tau)^2}{4Dt^2}\right), \tag{61}$$

$$\partial_t q(x_{\tau+t}|x_\tau) = \frac{1}{2\sqrt{\pi D t}} \exp\left[-\frac{(x_{\tau+t} - x_\tau)^2}{4Dt}\right] \left(-\frac{1}{2t} + \frac{(x_{\tau+t} - x_\tau)^2}{4Dt^2}\right). \tag{62}$$

Now we can evaluate each term in the square brackets in Supplementary Eq. (58) separately:

$$\left.\partial_t p(x_{\tau+t}|x_\tau) \ln \frac{p(x_{\tau+t}|x_\tau)}{q(x_{\tau+t}|x_\tau)}\right|_{t=0} = \frac{DF^2(x_\tau)}{2}\delta(x_{\tau+t} - x_\tau), \tag{63}$$

$$\left.\partial_t p(x_{\tau+t}|x_\tau)\right|_{t=0} = 0, \tag{64}$$

$$\left.\frac{p(x_{\tau+t}|x_\tau)}{q(x_{\tau+t}|x_\tau)} \partial_t q(x_{\tau+t}|x_\tau)\right|_{t=0} = \frac{DF^2(x_\tau)}{4}\delta(x_{\tau+t} - x_\tau), \tag{65}$$

so that Supplementary Eq. (58) evaluates to:

$$\lim_{\Delta t \to 0} \frac{S_{\mathrm{KL}}(\Delta t, \tau)}{\Delta t} = -\int dx_\tau p(x_\tau) dx_{\tau+t} \frac{DF^2(x_\tau)}{4} \delta(x_{\tau+t} - x_\tau) = -\frac{D}{4} \int dx_\tau F^2(x_\tau) p(x_\tau). \tag{66}$$

With this result, Supplementary Eq. (56) transforms into

$$S = S_{\mathrm{eq}} - \int_0^\infty d\tau \frac{D}{4} \int dx_\tau F^2(x_\tau) p(x_\tau, \tau), \tag{67}$$

which is equivalent to Eq. (14).

6.2 Numerical calculation of feature complexity

While the first term in Supplementary Eq. (67) can be evaluated in a straight-forward manner by numerical integration of Supplementary Eq. (54), the second term in Supplementary Eq. (67) involves space-time integral. We compute this term in the eigenbasis of the operator $\boldsymbol{\mathcal{H}}_0$ with the eigenvalues λ and the eigenfunctions $\Psi_0(x)$ defined by Supplementary

Eq. (3). Since Supplementary Eq. (3) is an eigenvalue-eigenvector problem, the solution for $\rho(x,t) = p(x,t)\exp(\Phi(x)/2)$ can be written as:

$$\rho(x,t) = \sum_k \rho_{0,k}\Psi_{0,k}(x)e^{-\lambda_k t}, \tag{68}$$

where $\rho_{0,k}$ are the coefficients of expansion of the function $\rho_0(x) = p_0(x)\exp(\Phi(x)/2)$ in the eigenbasis $\Psi_0(x)$. Using Supplementary Eq. (68), we obtain for the space-time integral:

$$\begin{aligned}
&\int_0^\infty dt \int_{-1}^1 dx p(x,t)F^2(x) = \int_0^\infty dt \int_{-1}^1 dx \rho(x,t)F^2(x)\exp[-\Phi(x)/2] = \\
&= \int_0^\infty dt \int_{-1}^1 dx \sum_k \Psi_{0,k}(x)\rho_{0,k}e^{-\lambda_k t}\sum_l \Psi_{0,l}(x)(F^2\exp[-\Phi/2])_l = \sum_k \frac{\rho_{0,k}(F^2\exp[-\Phi/2])_k}{\lambda_k},
\end{aligned} \tag{69}$$

where $(F^2\exp[-\Phi/2])_k$ are the coefficients of expansion of the function $F^2(x)\exp[-\Phi(x)/2]$ in the basis $\Psi_0(x)$. To derive Supplementary Eq. (69), we first used the eigenbasis expansion for the functions under the integral, and then we used the orthonormal properties of the eigenfunctions and evaluated the time integral analytically.

6.3 Numerical calculation of the Jensen-Shannon divergence

We approximate Eq. (15) by the midpoint rule:

$$D_{\text{JS}} = \Delta t \sum_{i=0}^{n} \text{JSD}\left(\hat{p}^1(x,t_i)||\hat{p}^2(x,t_i)\right), \tag{70}$$

where $t_i = i\Delta t$, and we choose the terminal time $t_{\max} = n\Delta t$ to be sufficiently large such that nearly all probability density $p(x,t)$ decays to zero due to absorption at the boundaries. Here $p(x,t)$ is the time-dependent solution of the Fokker-Planck equation Eq. (5).

Since JSD is defined for normalized distributions p and q, we take into account the probability loss through the absorbing boundaries and define a normalized distribution $\hat{p}(x,t) = p(x,t)+I_p\delta(x-x_b)$, where $I_p = 1-\int p(x,t)dx$ is the total probability loss through the absorbing boundaries up to time t. Here we do not distinguish between the probability loss through the left and right boundaries, and collapse the total probability loss into a single term. JSD is then calculated using the following expression:

$$\begin{aligned}
\text{JSD}(\hat{p}(x)||\hat{q}(x)) = &\frac{1}{2}\left(\int p(x)\log\frac{2p(x)}{p(x)+q(x)}dx + \int q(x)\log\frac{2q(x)}{p(x)+q(x)}dx\right) + \\
&+\frac{1}{2}\left(I_p\log\frac{2I_p}{I_p+I_q} + I_q\log\frac{2I_q}{I_p+I_q}\right),
\end{aligned} \tag{71}$$

where we used the sifting property of the delta function.

To calcuate D_{JS} between two models, we first solve the eigenvector-eigenvalue problem Supplementary Eq. (3) and use this solution to find the probability densities $p^1(x,t)$ and $p^2(x,t)$

via Supplementary Eq. (68) at time points $0, \Delta t, 2\Delta t, ..., n\Delta t$. Next, for each time point, we evaluate $I_p = 1 - \int p^1(x,t)dx$ and $I_q = 1 - \int p^2(x,t)dx$, and use Supplementary Eq. (71) to calculate JSD. Lastly, we sum JSDs across all time points using Supplementary Eq. (70). The numerical integration over the latent domain is performed by a dot product of the function with a vector of GLL weights (Supplementary Information 1).

7 Supplementary Table 1

Parameter	Value	Description
Ground-truth model parameters		
$\Phi(x)$	$-5x$	potential for ramping dynamics in Figs. 3,5, S2(a)
$\Phi(x)$	$-2.65x$	potential for ramping dynamics in Figs. 4,S2(b)
$\Phi(x)$	cubic splines*	potential for stepping dynamics (all figures)
$\Phi(x)$	$2(x/0.8)^4 - 4(x/0.8)^2$	double well potential in Fig. S1(a)
$\Phi(x)$	$2(x/0.6)^4 - 4(x/0.6)^2$	double well potential in Fig. S1(b,c)
D	0.1	noise for ramping dynamics in Figs. 3,5, S2(a)
D	0.56	noise for ramping dynamics in Figs. 4,S2(b)
D	1	noise for stepping dynamics
$p_0(x)$	$\propto \exp(-100(x+0.3)^2)$	initial probability density in Fig. 3,5,S2(a)
$p_0(x)$	$\propto \exp(-100x^2)$	initial probability density in Fig. 4,S1,S2(b,c)
$f(x)$	$50x + 60$	firing rate function, Hz
Spectral Elements Method		
x_{begin}	-1	left boundary in the latent space
x_{end}	1	right boundary in the latent space
N_v	111 — 447	number of eigenfunctions of $\boldsymbol{\mathcal{H}}$
N_e	16 — 64	number of elements
N_p	8	number of grid points per element
Optimization hyperparameters		
γ_F	0.001 — 0.005	learning rate for $F(x)$
γ_D	0.00025	learning rate for D
γ_{F_0}	0.01 — 0.025	learning rate for $F_0(x)$
$F^{(0)}(x)$	0	initialization for $F(x)$
$D^{(0)}$	1	initialization for D
$F_0^{(0)}(x)$	0	initialization for $F_0(x)$

Supplementary Table 1. Simulation parameters. *We define the potential function for stepping dynamics using six smoothly patched cubic splines. This potential shape is well approximated by the 14-degree polynomial: $\Phi(x) = 213.7x^{14} - 34.39x^{13} - 830.8x^{12} + 61.33x^{11} + 1329x^{10} + 37.88x^9 - 1144x^8 - 160.5x^7 + 590.7x^6 + 133x^5 - 192.4x^4 - 37.51x^3 + 33.03x^2 - 0.3233x + 0.4446$.

8 Supplementary Figures

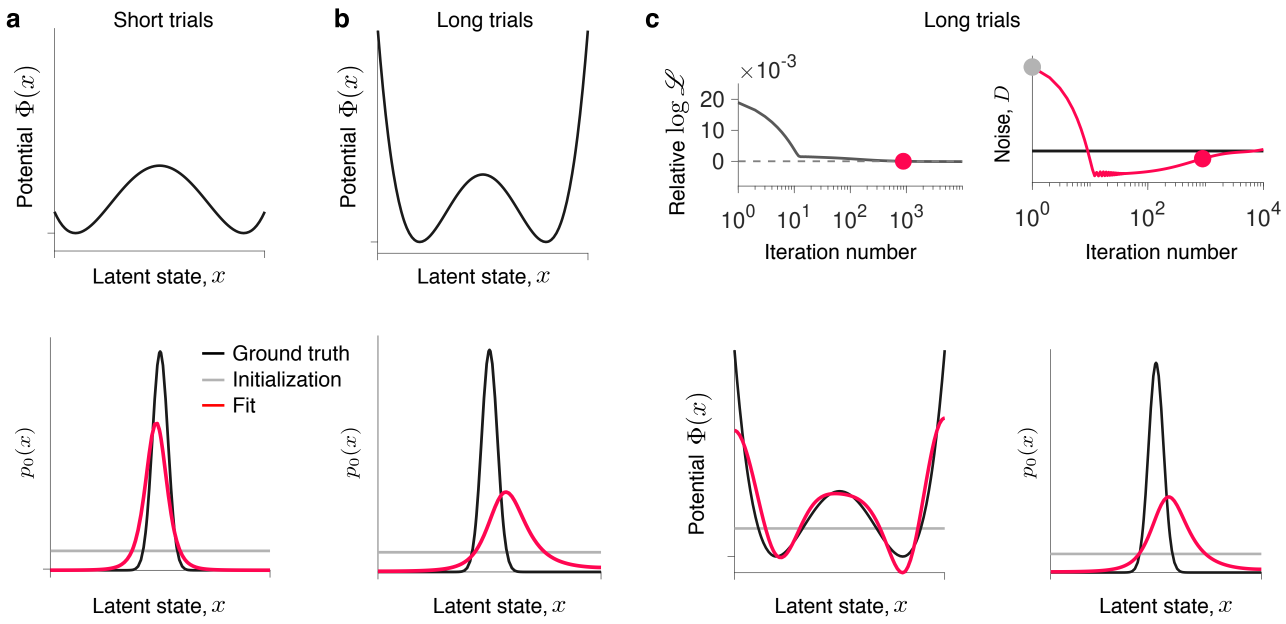

Supplementary Figure 1. The accuracy of inferring the initial state distribution depends on trial durations. (**a**) The initial state influences the trajectory only at short times before the dynamics equilibrate. When the data consist of many short trials such that the system has no time to equilibrate, the trajectories contain information about the initial state and $p_0(x)$ can be accurately inferred (lower panel). The data was generated from dynamics in a two-well potential (upper panel) with a narrow $p_0(x)$ distribution and absorbing boundary conditions. The low potential at the boundaries results in short trial durations. The data contained 100 trials with the average duration $\sim$ 1 second. (**b**) When the data consist of a few long trials such that the dynamics are largely at equilibrium, then trajectories contain little information about the initial state and the inference of $p_0(x)$ is less accurate (lower panel). The data was generated as in a but the potential has large barriers near the boundaries (upper panel), which results in long trial durations. The data contained 4 trials with the average duration $\sim$ 25 seconds, i.e. the total data amount is similar to a. (**c**) For long trials, the potential and noise magnitude can be inferred accurately even when the inference of $p_0(x)$ is inaccurate. The potential $\Phi(x)$, initial state distribution $p_0(x)$, and noise magnitude D are inferrred simultaneously from the same spike data as in b. For long trials, the dynamics are largely in equilibrium and independent of $p_0(x)$, and therefore accurate inference of $p_0(x)$ is not important for accurate inference of the potential function.

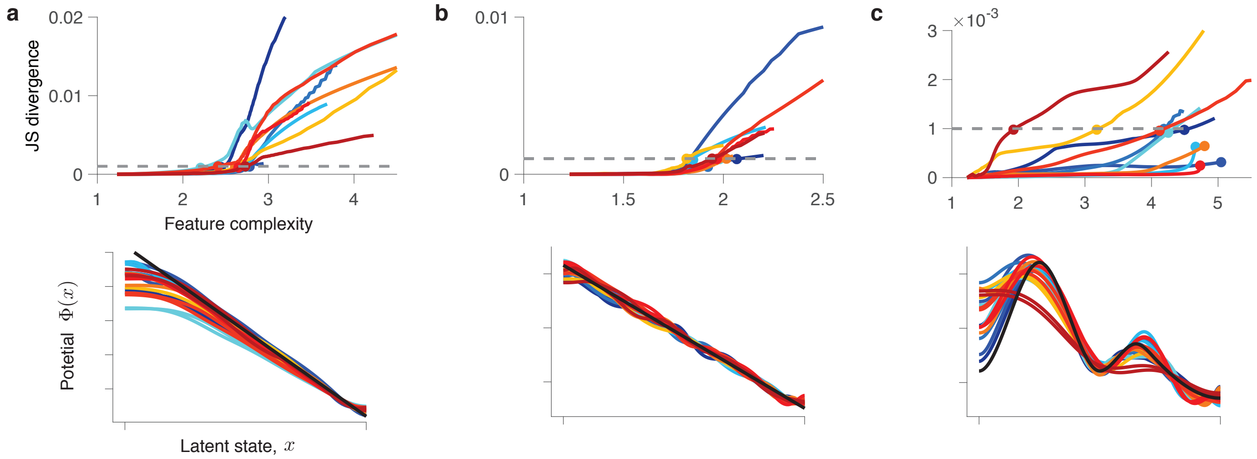

Supplementary Figure 2. Model selection based on feature consistency for ramping and stepping dynamics. Model selection based on feature consistency for (**a**) ramping dynamics with 400 trials, (**b**) ramping dynamics with 3,200 trials, and (**c**) stepping dynamics with 3,200 trials. For each model, we generated ten independent datasets and performed optimization and model selection on each dataset separately. We divide each dataset in two halves and compare features between the models fitted on each data half. We choose the optimal feature complexity $\mathcal{M}^*$ (upper row, color dots) by thresholding $D_{\mathrm{JS}}(\mathcal{M})$ at the value $D_{\mathrm{JS}}^{\mathrm{thres}} = 0.001$ (upper row, grey dashed line). For each of the ten datasets, the selected models (lower row, pairs of potentials shown with colored lines) agree well with the ground-truth (black line). For some of the simulations in c, the D_{JS} threshold was not reached after the maximum number of GD iterations (set to 20,000). In this case, we plotted the pair of models with the maximum achieved feature complexity.

9 Supplementary References

[1] Gardiner, C. W. *Handbook of Stochastic Methods*, vol. 3 (Springer Berlin, 1985).

[2] Genkin, M. & Engel, T. A. Moving beyond generalization to accurate interpretation of flexible models. *Nat. Mach. Intell.* **2**, 674–683 (2020).

[3] Haas, K. R., Yang, H. & Chu, J. W. Expectation-maximization of the potential of mean force and diffusion coefficient in langevin dynamics from single molecule fret data photon by photon. *J. Phys. Chem. B* **117**, 15591–15605 (2013).

[4] Deville, M. O., Fischer, P. F. & Mund, E. H. *High-order Methods for Incompressible Fluid Flow*, vol. 9 (Cambridge University Press, 2002).

[5] Bishop. *Pattern Recognition and Machine Learning* (Springer, 2006).

[6] Wilcox, R. M. Exponential operators and parameter differentiation in quantum physics. *J. Math. Phys.* **8**, 962–982 (1967).

[7] Dayan, P. & Abbott, L. F. *Theoretical neuroscience: computational and mathematical modeling of neural systems* (Computational Neuroscience Series, 2001).

[8] Paninski, L. Maximum likelihood estimation of cascade point-process neural encoding models. *Network: Computation in Neural Systems* **15**, 243–262 (2004).

[9] Øksendal, B. Stochastic differential equations. In *Stochastic differential equations*, 65–84 (Springer, 2003).

[10] Haas, K. R., Yang, H. & Chu, J.-W. Analysis of trajectory entropy for continuous stochastic processes at equilibrium. *The Journal of Physical Chemistry B* **118**, 8099–8107 (2014).

[11] Risken, H. *The Fokker-Planck Equation* (Springer, 1996).
